# Mortality According to CD4 Count at Start of Combination Antiretroviral Therapy Among HIV-infected Patients Followed for up to 15 Years After Start of Treatment: Collaborative Cohort Study

**DOI:** 10.1093/cid/ciw183

**Published:** 2016-03-29

**Authors:** Margaret T. May, Jorg-Janne Vehreschild, Adam Trickey, Niels Obel, Peter Reiss, Fabrice Bonnet, Murielle Mary-Krause, Hasina Samji, Matthias Cavassini, Michael John Gill, Leah C. Shepherd, Heidi M. Crane, Antonella d'Arminio Monforte, Greer A. Burkholder, Margaret M. Johnson, Paz Sobrino-Vegas, Pere Domingo, Robert Zangerle, Amy C. Justice, Timothy R. Sterling, José M. Miró, Jonathan A. C. Sterne

**Affiliations:** 1 Schoolof Social and Community Medicine, University of Bristol, United Kingdom; 2 Clinical Trials Unit II Infectious Diseases, University of Cologne, Germany; 3 Department of Infectious Diseases, Copenhagen University Hospital, Denmark; 4 Department of Global Health, Academic Medical Center, University of Amsterdam, and Amsterdam Institute of Global Health and Development HIV Monitoring Foundation; 5 Department of Internal Medicine, Division of Infectious Diseases, Center for Infection and Immunity–Amsterdam, Academic Medical Center, The Netherlands; 6 Bordeaux University, ISPED, INSERM U897; 7 CHU de Bordeaux; 8 Sorbonne Universités, UPMC Université Paris 06, INSERM, Institut Pierre Louis d’épidémiologie et de Santé Publique (IPLESP UMRS 1136), Paris, France; 9 Division of Epidemiology and Population Health, British Columbia Centre for Excellence in HIV/AIDS, Vancouver, Canada; 10 Service of Infectious Diseases, Lausanne University Hospital and University of Lausanne, Switzerland; 11 Division of Infectious Diseases, University of Calgary, Canada; 12 Research Department of Infection and Population Health, University College London Medical School, United Kingdom; 13 Center for AIDS Research, University of Washington, Seattle; 14 Monforte Clinic of Infectious Diseases and Tropical Medicine, San Paolo Hospital, University of Milan, Italy; 15 Division of Infectious Disease, Department of Medicine, University of Alabama, Birmingham; 16 Department of HIV Medicine, Royal Free London NHS Foundation Trust, United Kingdom; 17 Centro Nacional de Epidemiología, Instituto de Salud Carlos III, Madrid; 18 Department of Medicine, Autonomous University of Barcelona, Spain; 19 Medical University Innsbruck, Austria; 20 Yale University School of Medicine, New Haven; 21 Veterans Affairs Connecticut Healthcare System, West Haven, Connecticut; 22 Vanderbilt University School of Medicine, Nashville, Tennessee; 23 Hospital Clinic–IDIBAPS, University of Barcelona, Spain

**Keywords:** HIV, CD4 count, antiretroviral therapy, mortality, cohort collaboration

## Abstract

***Background.*** CD4 count at start of combination antiretroviral therapy (ART) is strongly associated with short-term survival, but its association with longer-term survival is less well characterized.

***Methods.*** We estimated mortality rates (MRs) by time since start of ART (<0.5, 0.5–0.9, 1–2.9, 3–4.9, 5–9.9, and ≥10 years) among patients from 18 European and North American cohorts who started ART during 1996–2001. Piecewise exponential models stratified by cohort were used to estimate crude and adjusted (for sex, age, transmission risk, period of starting ART [1996–1997, 1998–1999, 2000–2001], and AIDS and human immunodeficiency virus type 1 RNA at baseline) mortality rate ratios (MRRs) by CD4 count at start of ART (0–49, 50–99, 100–199, 200–349, 350–499, ≥500 cells/µL) overall and separately according to time since start of ART.

***Results.*** A total of 6344 of 37 496 patients died during 359 219 years of follow-up. The MR per 1000 person-years was 32.8 (95% confidence interval [CI], 30.2–35.5) during the first 6 months, declining to 16.0 (95% CI, 15.4–16.8) during 5–9.9 years and 14.2 (95% CI, 13.3–15.1) after 10 years’ duration of ART. During the first year of ART, there was a strong inverse association of CD4 count at start of ART with mortality. This diminished over the next 4 years. The adjusted MRR per CD4 group was 0.97 (95% CI, .94–1.00; *P* = .054) and 1.02 (95% CI, .98–1.07; *P* = .32) among patients followed for 5–9.9 and ≥10 years, respectively.

***Conclusions.*** After surviving 5 years of ART, the mortality of patients who started ART with low baseline CD4 count converged with mortality of patients with intermediate and high baseline CD4 counts.

## Abstract (precis)

The strong association of CD4 count at start of combination therapy with subsequent survival in HIV-infected patients diminished during the first 5 years of treatment. After 5 years, lower baseline CD4 counts were not associated with higher mortality.

Combination antiretroviral therapy (ART) based on ≥3 antiretroviral drugs including either a protease inhibitor (PI), nonnucleoside reverse transcriptase inhibitor (NNRTI), or integrase inhibitor has substantially improved the prognosis of human immunodeficiency virus type 1 (HIV-1)–infected patients since its introduction in high-income settings in 1996 [1]. ART suppresses HIV-1 replication, leading to declines in plasma HIV-1 RNA, increased CD4 T-cell counts, and, eventually, decreased morbidity and mortality [2]. Because patients have now been treated with ART for up to 20 years, it is of major interest to explore predictors of long-term prognosis.

The Antiretroviral Therapy Cohort Collaboration (ART-CC) was initiated in 2000 by investigators from HIV cohort studies in North America and Europe, to assemble datasets that were sufficiently large to study the prognosis of HIV-1–infected patients who started ART. Prognostic models that estimated 3- and 5-year survival were published in 2002 and 2007 [3, 4] and showed CD4 count at start of ART (baseline) to be the strongest predictor of short-term mortality. However, baseline CD4 count becomes less prognostic when 6-month response to treatment is taken into account [5]. HIV-1 RNA load at 6 months and changes in CD4 count from 6 to 36 months after start of ART are prognostic for AIDS in patients who have survived 3 years of ART [6].

To date, short-term prognosis has been extrapolated to predict long-term disease-free survival [7, 8]. Because mortality in the general population varies by age, sex, and country of residence, estimates of mortality rate ratios (MRRs) may be more informative when corrected for background mortality [9]. We investigated how long patients bear the burden of increased mortality due to starting ART with low CD4 counts, by studying the prognostic value of baseline CD4 count for mortality up to 15 years after start of ART, using both standard survival analysis models and models for relative survival.

## METHODS

### Study Sample

The ART-CC, which is described in detail elsewhere (http://www.art-cohort-collaboration.org) [10], is an international collaboration of HIV cohort studies from North America and Europe that combines data on HIV-infected individuals aged ≥16 years who were antiretroviral-naive when they started ART with a combination of at least 3 drugs. Eligible regimens include at least 1 PI, NNRTI, or integrase inhibitor and 2 nucleoside-analogue reverse transcriptase inhibitors (NRTIs). The present study included data from 18 cohorts, which are listed in the Supplementary Appendix. All were approved by local ethics committees. The National Health Service Health Research Authority South West–Cornwall and Plymouth Research Ethics Committee, United Kingdom, approved the study (REC reference 12/SW/0253). The data used here included follow-up from 1996 until 31 July 2013. We included patients who started ART between 1996 and 2001 (so had at least 12 years of potential follow-up) and had at least 1 available baseline (within 3 months prior to starting ART) measurement of CD4 count and viral load.

### Statistical Analysis

We categorized CD4 counts into 6 groups (0–49, 50–99, 100–199, 200–349, 350–499, and ≥500 cells/µL); viral loads into 3 groups (0–9999, 10 000–99 999, and ≥100 000 copies/µL); age at baseline into 5 groups (16–29, 30–39, 40–49, 50–59, and ≥60 years); year of starting ART into 3 groups (1996–1997, 1998–1999, and 2000–2001); and ART regimen into 5 groups (NNRTI-based, PI-based, NRTI including abacavir, other NRTI not including abacavir, and other regimens). Patients were followed up from the date of starting ART (“baseline”) to the earliest of the date of death, loss to follow-up, or administrative censoring. Patients were considered lost to follow-up at their last clinical observation if it was more than a year before the cohort-specific close date of the database. Data were analyzed as intent to continue treatment, ignoring treatment changes and interruptions.

We used unadjusted (Kaplan–Meier) and adjusted estimates of cumulative mortality to examine whether CD4 count at start of ART was prognostic for mortality. The adjusted estimates were for a typical patient: men who have sex with men (MSM) aged 30–39 years, who started ART between 1998 and 1999 in the French Hospital Database on HIV (FHDH) cohort without a diagnosis of AIDS, and with high viral load (HIV-1 RNA > 100 000 copies/µL). We used Poisson models to estimate crude mortality rates (MRs) with 95% confidence intervals (CIs), for all patients and by baseline CD4 count. We then estimated MRs separately according to duration of time on ART (<0.5, 0.5–0.9, 1–2.9, 3–4.9, 5–9.9, and ≥10 years after ART start), overall, and by baseline CD4 count. We used Cox models stratified by cohort to estimate crude and adjusted (for sex, age, transmission risk group, AIDS at baseline, baseline viral load, and year of starting ART) MRRs by baseline CD4 group with 200–349 cells/µL as the reference category, overall and separately according to time on ART. Relative survival models based on generalized linear models (modified Poisson models) were used to account for variation in background population mortality [11]. Expected death rates for each country split by sex and 5-year age group were obtained from the Human Mortality Database (www.mortality.org). We estimated adjusted (for the same set of covariates) relative MRR, overall and according to duration of time on ART. The prognostic value of baseline CD4 count according to time since start of ART was compared by estimating the adjusted MRR per CD4 count category (assuming a log-linear relationship with MR across the CD4 categories). All analyses were performed using Stata software version 13 (StataCorp, College Station, Texas).

## RESULTS

A total of 37 496 patients started ART between 1996 and 2001 and were eligible for analyses. The majority were male, with a median age at start of ART of 37 years (interquartile range [IQR], 31–44 years; Table 1). Median follow-up time was 11.3 years (IQR, 5.6–13.4 years), with 28 947 (77%) of patients having at least 5 years and 21 936 (58%) >10 years' follow-up. Numbers of patients at risk by duration of ART and baseline CD4 count are shown in Supplementary Table 1. The risk transmission group distribution reflected the early HIV epidemic, with similar proportions of MSM and heterosexually infected patients, and 20% infected via injection drug use. The median baseline CD4 count was 221 cells/µL (IQR, 86–376 cells/µL): 17 077 (46%) patients started ART with CD4 count <200 cells/µL and 10 278 (27%) with CD4 count <100 cells/µL. Compared with patients who started ART with CD4 count <350 cells/µL, those who started with higher CD4 count were more likely to be MSM and to have started ART in 1997–1999, and were slightly younger on average. The majority of patients (24 313 [65%]) started on a PI-based regimen, whereas 10 146 (27%) started on an NNRTI-based regimen. The rate of loss to follow-up was 0.04 per year.

**Table 1.** Demographic and Clinical Characteristics at Start of Antiretroviral Therapy (N = 37 496)

Characteristic	No. of Patients (%)	No. of Deaths (%)
Year of starting ART		
1996–1997	8304 (22)	1550 (19)
1998–1999	15 599 (42)	2760 (18)
2000–2001	13 593 (36)	2034 (15)
Female sex	8293 (22)	817 (10)
AIDS at start of ART	8516 (23)	2103 (25)
CD4 count, cells/µL		
Median (IQR)	221 (86–376)	
0–49	6512 (17)	1654 (25)
50–99	3766 (10)	844 (22)
100–199	6799 (18)	1292 (19)
200–349	9633 (26)	1338 (14)
350–499	5990 (16)	697 (12)
≥500	4796 (13)	519 (11)
HIV-1 RNA, copies/µL		
Median (IQR)	75 000 (18 000–239 000)	
0–9999	6834 (18)	843 (12)
10 000–99 999	14 149 (38)	2 186 (15)
≥100 000	16 513 (44)	3315 (20)
Age, y		
Median (IQR)	37 (31–44)	
16–29	6734 (18)	514 (8)
30–39	16 572 (44)	2094 (13)
40–49	9104 (24)	1989 (22)
50–59	3766 (10)	1184 (31)
≥60	1320 (4)	563 (43)
Risk transmission group		
MSM	11 067 (30)	1056 (10)
Injection drug use	7626 (20)	1625 (21)
Heterosexual	11 709 (31)	1122 (10)
Blood	440 (1)	77 (18)
Other or unknown	6654 (18)	2464 (37)
Length of follow-up, y, median (IQR)	11.3 (5.6–13.4)	
Regimen		
NNRTI-based	10 146 (27)	1559 (15)
PI-based	24 313 (65)	4371 (18)
Triple NRTI (including abacavir)	2006 (5)	240 (12)
Other-NRTI	760 (2)	112 (15)
Other	271 (1)	62 (23)

Abbreviations: ART, antiretroviral therapy; HIV-1, human immunodeficiency virus type 1; IQR, interquartile range; MSM, men who have sex with men; NNRTI, nonnucleoside reverse transcriptase inhibitor; NRTI, nucleoside-analogue reverse transcriptase inhibitor; PI, protease inhibitor.

**Figure 1.**
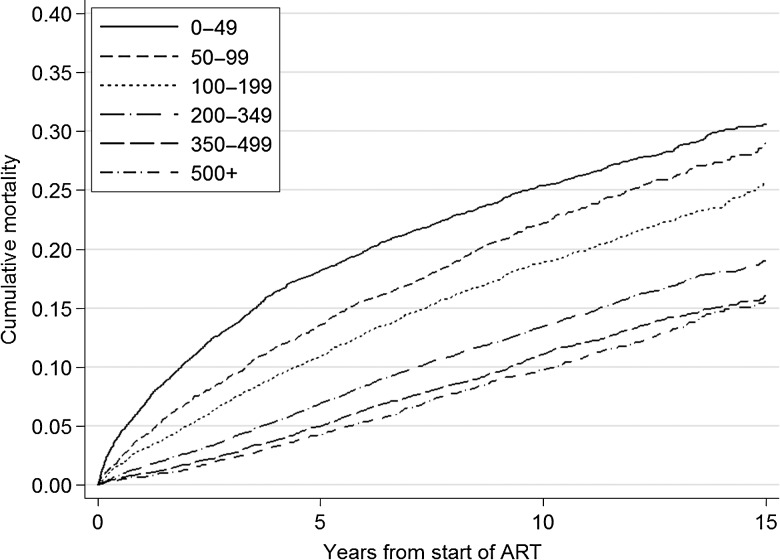
Unadjusted estimates of overall cumulative mortality by CD4 cell count at start of antiretroviral therapy (ART).

**Figure 2.**
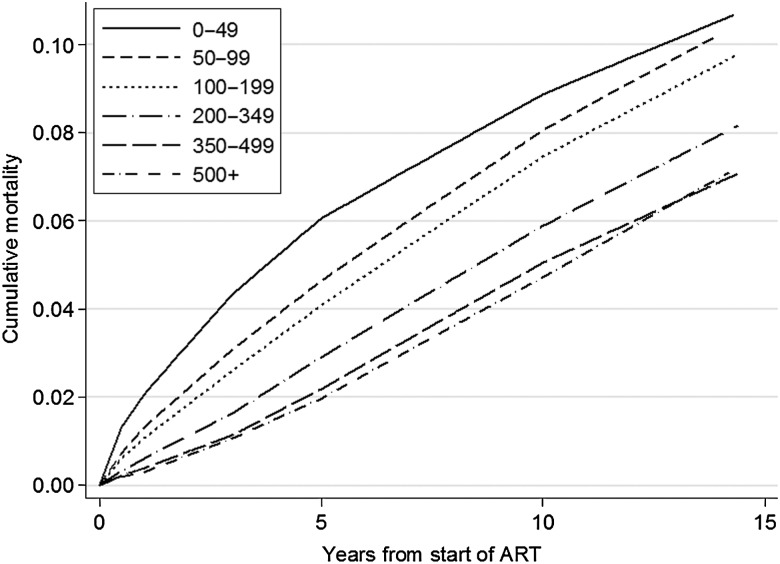
Adjusted estimates of cumulative mortality by CD4 cell count at start of antiretroviral therapy (ART) for a typical patient group (men who have sex with men, aged 30–39 years, who started ART between 1998 and 1999 without a diagnosis of AIDS, with high viral load [human immunodeficiency virus {HIV}-1 RNA > 100 000 copies/µL]) from the French Hospital Database on HIV cohort.

There were 6344 deaths during 359 219 years of follow-up. Baseline CD4 count was highly prognostic for unadjusted cumulative mortality in all patients (Figure 1). Baseline CD4 count was also highly prognostic for adjusted cumulative mortality, which is illustrated for a typical patient group (MSM in the FHDH cohort, aged 30–39 years, who started ART between 1998 and 1999 without a diagnosis of AIDS, and with high viral load [HIV-1 RNA > 100 000 copies/µL]) in Figure 2. There is a clearly graded increase in mortality with decreasing baseline CD4 count: Cumulative 15-year mortality varied from 7.1% (95% CI, 2.9%–11.3%) for baseline CD4 count >500 cells/µL to 10.7% (95% CI, 4.7%–16.7%) for baseline CD4 count <50 cells/µL. A table showing the corresponding numbers at risk for Figure 1 at different time-points split by CD4 count is available from the authors on request.

Numbers of deaths and crude MRs per 1000 person-years, according to baseline CD4 count and time since start of ART, are shown in Table 2. Overall mortality per 1000 person-years declined from 33 (95% CI, 30–36) during the first 6 months of ART to 14 (95% CI, 13–15) among those who survived 10 years from start of ART, despite the aging of the study population over this period. Patients who started ART with a CD4 count <50 cells/µL experienced the greatest declines in MRs over time on ART, from 88 (95% CI, 78–99) per 1000 person-years during the first 6 months to 15 (95% CI, 13–17) per 1000 person-years after 10 years from start of ART. Although MRs were strongly inversely associated with baseline CD4 counts at shorter times since start of ART, the magnitude of these associations diminished with increasing duration of ART. Beyond 10 years of ART, there was little evidence of differences in crude MRs according to CD4 count at start of ART.

**Table 2.** Numbers of Deaths (n = 6344) and Crude Mortality Rates per 1000 Person-years, by CD4 Count at Start of Antiretroviral Therapy and Duration of Follow-up

	Duration of Follow-up From Start of ART, y											
	<0.5 (n = 37 496)		0.5–0.9 (n = 35 928)		1–2.9 (n = 34 841)		3–4.9 (n = 31 185)		5–9.9 (n = 28 944)		≥10 (n = 21 931)	
CD4 Count at ART Start, Cells/µL	No. of Deaths	MR (95% CI)	No. of Deaths	MR (95% CI)	No. of Deaths	MR (95% CI)	No. of Deaths	MR (95% CI)	No. of Deaths	MR (95% CI)	No. of Deaths	MR (95% CI)
0–49	275	88 (78.2–99.0)	145	48.9 (41.6–57.5)	414	38.1 (34.6–42.0)	275	28.4 (25.3–32.0)	381	18.6 (16.8–20.6)	164	14.8 (12.7–17.2)
50–99	84	45.8 (37.0–56.7)	64	36.5 (28.5–46.6)	181	27.9 (24.1–32.2)	143	24.4 (20.7–28.8)	258	21.1 (18.7–23.9)	114	17.8 (14.8–21.4)
100–199	115	34.7 (28.9–41.7)	82	25.7 (20.7–31.9)	261	21.9 (19.4–24.7)	224	20.7 (18.2–23.6)	426	18.9 (17.2–20.8)	184	15.8 (13.7–18.3)
200–349	75	15.9 (12.6–19.9)	62	13.5 (10.5–17.4)	221	12.7 (11.2–14.5)	248	15.5 (13.7–17.6)	490	14.6 (13.4–16.0)	242	13.6 (12.0–15.4)
350–499	30	10.2 (7.1–14.6)	22	7.7 (5.0–11.6)	96	8.8 (7.2–10.7)	122	12 (10.1–14.4)	288	13.3 (11.9–14.9)	139	11.5 (9.7–13.6)
≥500	20	8.5 (5.4–13.2)	10	4.3 (2.3–8.1)	73	8.4 (6.6–10.5)	79	9.8 (7.9–12.2)	206	11.9 (10.4–13.6)	131	13.5 (11.4–16.1)
All	599	32.8 (30.2–35.5)	385	21.8 (19.7–24.1)	1246	18.8 (17.8–19.9)	1091	18.0 (17.0–19.1)	2049	16.0 (15.4–16.8)	974	14.2 (13.3–15.1)

Abbreviations: ART, antiretroviral therapy; CI, confidence interval; MR, mortality rate.

Table 3 shows associations of CD4 count at start of ART with mortality at different durations since start of ART and averaged over all follow-up time, estimated from crude, adjusted, and relative survival models. Estimates are presented as MRRs with 95% CIs, with CD4 count 200–349 cells/µL as the comparator group. As expected crude, adjusted, and relative MRRs averaged over all follow-up time decreased with increasing CD4 count at start of ART. After adjusting for other prognostic factors, the MRR comparing baseline CD4 count <50 cells/µL with 200–349 cells/µL during the first 6 months of ART was 2.81 (95% CI, 2.12–3.71): this declined to 1.59 (95% CI, 1.31–1.92) 3–4.9 years after start of ART. During the first 5 years of ART, rate ratios comparing patients whose baseline CD4 count was >350 cells/µL with those whose count was 200–349 cells/µL were <1. However, there was little evidence that baseline CD4 count was prognostic for mortality after 5 years of ART as the adjusted MRRs per CD4 category were 0.97 (95% CI, .94–1.00; *P* = .054) and 1.02 (95% CI, .98–1.07; *P* = .32) among patients followed for 5–9.9 and ≥10 years, respectively. Adjusted relative MRRs were generally further from 1 than the corresponding adjusted MRRs, indicating that failing to account for expected mortality partially obscured the association of CD4 count with mortality risk. Differences between the 2 analyses diminished with increasing time on ART because the strength of the association of mortality with baseline CD4 count also decreased with time.

**Table 3.** Crude and Adjusted Associations of CD4 Cell Count at Start of Antiretroviral Therapy (ART) With Mortality at Increasing Durations Since Start of ART

Duration of ART, y	CD4 Count, Cells/µL					
	0–49	50–99	100–199	200–349	350–499	≥500
	n = 6512 (1654 Deaths)	n = 3766 (844 Deaths)	n = 6799 (1292 Deaths)	n = 9633 (1338 Deaths)	n = 5990 (697 Deaths)	n = 4796 (519 Deaths)
Crude MRR (95% CI)						
<0.5	5.51 (4.26–7.12)	2.92 (2.14–3.99)	2.22 (1.66–2.97)	1	0.63 (.41–.96)	0.53 (.32–.87)
0.5–0.9	3.47 (2.57–4.68)	2.71 (1.91–3.84)	1.92 (1.38–2.67)	1	0.56 (.34–.91)	0.32 (.17–.63)
1–2.9	2.85 (2.42–3.36)	2.16 (1.78–2.63)	1.72 (1.44–2.06)	1	0.69 (.54–.86)	0.67 (.51–.87)
3–4.9	1.72 (1.45–2.05)	1.55 (1.26–1.90)	1.34 (1.12–1.61)	1	0.78 (.63–.97)	0.65 (.51–.84)
5–9.9	1.17 (1.02–1.34)	1.42 (1.22–1.65)	1.30 (1.15–1.49)	1	0.92 (.80–1.07)	0.83 (.71–.98)
≥10	1.03 (.84–1.25)	1.31 (1.05–1.64)	1.17 (.97–1.42)	1	0.84 (.68–1.03)	0.98 (.79–1.21)
All time	1.87 (1.74–2.02)	1.69 (1.55–1.84)	1.44 (1.33–1.55)	1	0.81 (.74–.89)	0.77 (.69–.85)
Adjusted MRR (95% CI)						
<0.5	2.81 (2.12–3.71)	1.71 (1.24–2.37)	1.67 (1.25–2.25)	1	0.70 (.46–1.07)	0.62 (.38–1.02)
0.5–0.9	2.50 (1.80–3.47)	2.04 (1.41–2.93)	1.65 (1.18–2.31)	1	0.59 (.36–.96)	0.35 (.18–.69)
1–2.9	2.33 (1.95–2.79)	1.76 (1.44–2.16)	1.53 (1.28–1.83)	1	0.72 (.57–.91)	0.72 (.55–.94)
3–4.9	1.59 (1.31–1.92)	1.36 (1.10–1.69)	1.22 (1.02–1.47)	1	0.80 (.64–1.00)	0.70 (.55–.91)
5–9.9	1.01 (.87–1.17)	1.20 (1.02–1.40)	1.16 (1.02–1.32)	1	0.96 (.83–1.11)	0.92 (.78–1.08)
≥10	0.85 (.68–1.05)	1.07 (.85–1.35)	1.01 (.83–1.22)	1	0.88 (.71–1.09)	1.09 (.88–1.35)
All time	1.51 (1.40–1.64)	1.37 (1.25–1.50)	1.26 (1.16–1.36)	1	0.85 (.78–.93)	0.84 (.76–.93)
Adjusted relative MRR (95% CI)						
<0.5	3.15 (2.28–4.36)	1.87 (1.29–2.72)	1.84 (1.31–2.59)	1	0.66 (.39–1.11)	0.52 (.27–1.02)
0.5–0.9	2.91 (1.97–4.31)	2.38 (1.55–3.65)	1.80 (1.20–2.70)	1	0.46 (.22–.94)	0.30 (.12–.75)
1–2.9	2.78 (2.23–3.48)	2.01 (1.57–2.58)	1.66 (1.32–2.09)	1	0.65 (.47–.91)	0.65 (.45–.93)
3–4.9	1.74 (1.39–2.20)	1.42 (1.09–1.85)	1.31 (1.04–1.63)	1	0.78 (.59–1.03)	0.63 (.45–.90)
5–9.9	0.99 (.81–1.21)	1.27 (1.03–1.56)	1.20 (1.00–1.43)	1	0.94 (.77–1.14)	0.79 (.63–1.00)
≥10	0.79 (.55–1.15)	0.98 (.65–1.47)	1.01 (.73–1.40)	1	0.79 (.55–1.14)	1.12 (.79–1.58)
All time	1.72 (1.56–1.91)	1.51 (1.35–1.70)	1.35 (1.22–1.50)	1	0.80 (.70–.91)	0.75 (.64–.86)

The relative survival model accounts for age-, sex-, and country-matched population mortality. Models were stratified by cohort. Adjusted and relative survival models were adjusted for sex, age, transmission risk group, AIDS at baseline, viral load, and year of starting ART.

Abbreviations: ART, antiretroviral therapy; CI, confidence interval; MRR, mortality rate ratio.

## DISCUSSION

In our collaborative analysis of 37 496 HIV-infected patients from 18 cohorts starting triple-combination ART between 1996 and 2001, there was a strong inverse association of CD4 count with mortality during the first year of ART, which diminished over the next 4 years. From 5 years after start of ART, baseline CD4 count was of little prognostic value. Even patients who started ART with very low (<50 cells/µL) CD4 counts may experience convergence of their mortality risk to that of patients with intermediate (200–349 cells/µL) or high (≥500 cells/µL) baseline CD4 count, from 5 years after the start of treatment. As expected, associations of baseline CD4 count with mortality were attenuated after adjusting for other prognostic factors at the time of starting ART. Associations moved away from the null after further adjusting for background mortality in relative survival models, but this did not impact our results substantially.

The importance of CD4 count nadir as a prognostic factor for survival in HIV-infected individuals starting ART is well established [3]. However, the extent to which patients with low baseline CD4 count surviving the first years of treatment remain at an increased risk of death, compared to patients starting with higher baseline CD4 counts, has remained unclear. To our knowledge, our study is the first to demonstrate that patients who started treatment in later stages of HIV disease may expect their mortality risk to become similar to that of patients starting treatment with higher CD4 counts, if they survive the first 5 years of therapy. The majority of patients (85.7% [95% CI, 85.2%–86.3%]) who started ART with CD4 count <200 cells/µL in this cohort collaboration do indeed survive 5 years. This is an important message for patients and physicians alike, as the moving from a higher- to a lower-risk group through long-term treatment adherence may be a powerful motivator. Our findings are consistent with previous studies which showed that current CD4 count is more important than baseline CD4 count [5, 6].

For the first 5 years of ART, there was a graded inverse relationship between baseline CD4 count and mortality, which was consistent with our previous findings [12]. After 5 years of ART, >15% of the patients with a baseline CD4 count of <50 cells/µL had died. These results are consistent with the rate of immunological nonresponders to ART previously observed [13, 14]. Patients with poor immunological response or multiple AIDS or non-AIDS morbidity continue to have a substantially increased mortality ratio after 3 years of treatment [14, 15]. A low CD4 nadir is not only an established risk factor for death, but also for poor CD4 recovery [16–18]. This may reach a plateau after about 2 years of ART [18], although a very recent analysis indicates slow but steady long-term recovery of CD4 counts under continued treatment [15].

These findings raise the question of how to identify patients at high risk of mortality during the first 5 years of ART and what strategies could reduce this risk. These could include screening programs and intensive adherence counseling. A previous study demonstrated that patients with a CD4 count<200 cells/µL at baseline are at a higher risk for AIDS in particular, but also for non-AIDS-related mortality [12]. In that study, malignancies and infections were the primary causes of death, and both occurred more frequently in patients with a CD4 count <200 cells/µL. The observed convergence of survival rates after 5 years may be partly explained by the early death of patients with low to very low baseline CD4 count, especially immunological nonresponders, whereas others gradually achieve immunological recovery. The suggestion of a lower risk among those who started ART with very low compared with intermediate CD4 count after 10 years may reflect a cohort of survivors who are adherent and respond well to therapy, but is also consistent with a chance finding.

We analyzed large numbers of patients from high-quality clinical cohorts. We obtained more accurate comparisons by using relative survival models that adjust for both patient characteristics and the background age, sex, and country general population mortality. Adjusting associations of CD4 count with mortality for age and sex as covariates in a conventional analysis that only considers mortality of those with HIV infection may be inducing bias toward the null. Although additionally adjusting for background mortality increased associations of baseline CD4 count with both cumulative mortality and early mortality, it did not affect our conclusions about the convergence of mortality in those who survived 5 years from starting ART.

Our study has some limitations. We chose patients starting ART between 1996 and 2001 to allow ≥10 years of follow-up for each person included. Whereas only such patients have been treated with long-term ART, newer drugs that have been introduced since 2002 are associated with better immune recovery [19] and/or CD4 recovery in immunological nonresponders [19–21], compared with drugs on which the patients in our study started combination ART. More second-line treatment options and new formulations with improved convenience have become available, allowing better treatment adherence and successful treatment of drug-resistant infections. Therefore, patients across all baseline CD4 count strata are likely to experience lower mortality than that of the patients included in our study.

While we included a broad range of patients with different risk factors from Europe and North America, generalizability to patients treated in other settings may be limited. Our results may be biased by loss to follow-up. Although loss to follow-up rates were high, cohorts have good ascertainment of death procedures and it is therefore more likely that patients transferred care to another center not in ART-CC or dropped out of care, rather than died [22]. However, our results should be generalizable to individuals remaining in HIV care. The high death rate observed in patients with unknown risk transmission is linked to the high death rate in older patients because risk group was poorly recorded in cohorts that included more patients aged >60 years. Data on non-HIV-related factors (eg, socioeconomic disadvantage or lifestyle risk factors) were not available, but may have contributed to excess mortality during the first 5 years of treatment in patients with low baseline CD4 counts.

In conclusion, CD4 count at start of ART strongly predicts MRs during the first 5 years of ART. This finding reinforces the need for earlier diagnosis and treatment of people living with HIV. However, there is little evidence that CD4 count at start of ART predicts mortality after 5 years of ART. This is a positive message for patients: The burden of increased mortality associated with starting treatment late is alleviated for those who survive 5 years on ART.

## Supplementary Data

Supplementary materials are available at http://cid.oxfordjournals.org. Consisting of data provided by the author to benefit the reader, the posted materials are not copyedited and are the sole responsibility of the author, so questions or comments should be addressed to the author.

Supplementary Data

## APPENDIX

***Cohorts contributing to this analysis.*** French Hospital Database on HIV (FHDH); the Italian Cohort of Antiretroviral-Naive patients (ICONA); the Swiss HIV Cohort Study (SHCS); the AIDS Therapy Evaluation project, Netherlands (ATHENA); the Aquitaine Cohort; the Royal Free Hospital Cohort, UK; the South Alberta Clinic Cohort; 1917 Clinic Cohort, University of Alabama at Birmingham; The Danish HIV Cohort Study, Denmark; HAART Observational Medical Evaluation and Research (HOMER), Canada; HIV Atlanta Veterans Affairs Cohort Study (HAVACS); Osterreichische HIV-Kohortenstudie (OEHIVKOS), Austria; Proyecto para la Informatizacion del Seguimiento Clinico-epidemiologico de la Infeccion por HIV y SIDA (PISCIS), Spain; University of Washington HIV Cohort; VACH, Spain; Veterans Aging Cohort Study (VACS); Vanderbilt University; and the Koln/Bonn Cohort.

***ART-CC Steering Group.*** Andrew Boulle (IeDEA Southern Africa), Christoph Stephan (Frankfurt), Jose M. Miro (PISCIS), Matthias Cavassini (SHCS), Geneviève Chêne (Aquitaine), Dominique Costagliola (FHDH), François Dabis (Aquitaine), Antonella D'Arminio Monforte (ICONA), Julia del Amo (CoRIS-MD), Ard Van Sighem (ATHENA), Gerd Fätkenheuer (Koln/Bonn), John Gill (South Alberta Clinic), Jodie Guest (HAVACS), David Hans-Ulrich Haerry (EATG), Robert Hogg (HOMER), Amy Justice (VACS), Leah Shepherd (EuroSIDA), Neils Obel (Denmark), Heidi Crane (Washington), Colette Smith (Royal Free), Peter Reiss (ATHENA), Michael Saag (Alabama), Tim Sterling (Vanderbilt-Meherry), Ramon Teira (VACH), Matthew Williams (UK-CAB), and Robert Zangerle (Austria).

***ART-CC Coordinating Team.*** Jonathan Sterne and Margaret May (principal investigators), Suzanne Ingle, Adam Trickey (statisticians).
