# Supplementary material for: Mortality According to CD4 Count at Start of Combination Antiretroviral Therapy Among HIV-infected Patients Followed for up to 15 Years After Start of Treatment: Collaborative Cohort Study
Source: Clin Infect Dis. 2016 Mar 29;62(12):1571–7. doi: 10.1093/cid/ciw183 (PMC4885653; doi:10.1093/cid/ciw183)
Supplement: Supplementary Data [file supp_62_12_1571__index.html]

Mortality according to CD4 count at start of combination antiretroviral therapy among HIV positive patients followed for up to 15 years after start of treatment: collaborative cohort study — Mortality According to CD4 Count at Start of Combination Antiretroviral Therapy Among HIV-infected Patients Followed for up to 15 Years After Start of Treatment: Collaborative Cohort Study — Mortality According to CD4 Count at Start of Combination Antiretroviral Therapy Among HIV-infected Patients Followed for up to 15 Years After Start of Treatment: Collaborative Cohort Study — Supplementary Data 

# Mortality According to CD4 Count at Start of Combination Antiretroviral Therapy Among HIV-infected Patients Followed for up to 15 Years After Start of Treatment: Collaborative Cohort Study

## Supplementary Data

Supplementary Data

- Supplementary Data - Docx file
