# Supplementary material for: Mortality According to CD4 Count at Start of Combination Antiretroviral Therapy Among HIV-infected Patients Followed for up to 15 Years After Start of Treatment: Collaborative Cohort Study
Source: Clin Infect Dis. 2016 Mar 29;62(12):1571–7. doi: 10.1093/cid/ciw183 (PMC4885653; doi:10.1093/cid/ciw183)
Supplement: Supplementary Data [file supp_ciw183_ciw183supp.docx]

# Mortality according to CD4 count at start of combination antiretroviral therapy among HIV positive patients followed for up to 15 years after start of treatment: collaborative cohort study

**T**he Antiretroviral Therapy Cohort Collaboration (ART-CC)

Supplementary table

**Web table 1: Numbers of patients at risk by duration of ART and baseline CD4 count.**

| Baseline CD4 count (cells/µl) | Duration of follow up after ART start (years) | | | |
| --- | --- | --- | --- | --- |
|  | 0 | 5 | 10 | 15 |
|  | Number of patients remaining in follow-up | | | |
| 0-49 | 6,512 | 4,612 | 3,541 | 541 |
| 50-99 | 3,766 | 2,790 | 2,083 | 316 |
| 100-199 | 6,799 | 5,154 | 3,849 | 588 |
| 200-349 | 9,633 | 7,635 | 5,725 | 876 |
| 350-499 | 5,990 | 4,870 | 3,724 | 600 |
| ≥500 | 4,796 | 3,881 | 3,009 | 470 |
| **All** | **37,496** | **28,947** | **21,936** | **3,415** |
